# The occurrence of seizures after ischemic stroke does not influence long-term mortality; a 26-year follow-up study

**DOI:** 10.1007/s00415-018-8907-7

**Published:** 2018-05-29

**Authors:** J. H. van Tuijl, E. P. M. van Raak, R. J. van Oostenbrugge, A. P. Aldenkamp, R. P. W. Rouhl

**Affiliations:** 1 Department of Neurology, Maastricht University Medical Center, PO Box 5800, 6202 AZ Maastricht, The Netherlands; 2 Department of Neurology, Elisabeth-Tweesteden Hospital, Tilburg, The Netherlands; 3 School for Mental Health and Neurosciences, Maastricht University, Maastricht, The Netherlands; 4 Cardiovascular Research Institute Maastricht (CARIM), Maastricht University Medical Center, Maastricht, The Netherlands; 5 Academic Center for Epileptology, Maastricht University Medical Center and Kempenhaeghe Center of Expertise for Epileptology, Maastricht, The Netherlands; 6 Faculty of Electrical Engineering, University of Technology, Eindhoven, The Netherlands

**Keywords:** Seizures, Epilepsy, Brain infarct, Stroke

## Abstract

**Objective:**

Epileptic seizures are a common complication after stroke. The relation between occurrence of seizures after stroke and long-term mortality remains elusive. We aimed to assess whether seizures in an early or late phase after ischemic stroke are an independent determinant of long-term mortality.

**Methods:**

We prospectively included and followed 444 ischemic stroke patients with a first-ever supratentorial brain infarct for at least 2 years after their stroke regarding the occurrence of seizures. The final follow-up for mortality is from April 2015 (follow-up duration 24.5–27.8 years, mean 26.0 years, SD 0.9 years). We compared patients with early-onset seizures with all seizure-free patients, whereas the patients with late-onset seizures were compared with the 1-week survivors without any seizures. We used Cox-regression analyses to correct for possible confounding factors.

**Results:**

Kaplan–Meier analysis showed significantly higher mortality for the patients with early-onset seizures (*p* = 0.002) but after correction for known risk factors for (long term) mortality early-onset seizures had no independent influence on long-term mortality (HR 1.09; 95% CI 0.64–1.85). In patients with late-onset seizures, no significant influence from late-onset seizures on long-term mortality was found (univariate *p* = 0.717; multivariate HR 0.81; 95% CI 0.54–1.20).

**Conclusion:**

Both early-onset and late-onset seizures do not influence long-term mortality after ischemic stroke.

## Introduction

Epileptic seizures are a common complication after stroke although the risk to develop seizures varies, depending on variables such as stroke type (hemorrhagic vs. ischemic), stroke severity, and cortical involvement [1–7]. Seizures occurring after stoke are divided in early-onset seizures (ES, incidence 3.3% of patients) and late-onset seizures (LS, incidence 18 in 1000 person years) depending on their time of occurrence, with a variable cut-off point in different studies between 24 h and 1 month post-stroke [2, 6, 8, 9]. ES and LS differ in their pathophysiology. ES are induced by the acute disruption of the blood–brain barrier and the resultant hyperexcitatory state, whereas LS are a result of a chronic process called epileptogenesis, which causes gliosis and formation of epileptogenic networks [10]. Furthermore, LS have a higher recurrence rate than ES [11] and, therefore, LS are considered to have a larger clinical significance. In addition, according to the guidelines of the International League for Epilepsy (ILAE), in stroke patients with LS, epilepsy may be diagnosed after only one seizure [12].

In general, patients with epilepsy have a higher mortality risk than people without epilepsy [13]. However, whether this also applies to stroke patients suffering from post-stroke seizures remains unclear. Most studies showed a negative relation between ES and mortality when using a univariate statistical analysis technique; however, this relationship was lost when using multivariate statistical analysis techniques [14–18]. Only in two studies by Arboix et al. the relationship between early seizures and mortality remained significant [19, 20]. We are aware of only one study that studied the relation between LS and mortality in stroke patients [21]. This study in young stroke patients showed that long-term mortality was significantly higher in patients with seizures than in patients without seizures.

Knowledge of the impact of especially late-onset seizures on (long term) mortality in stroke patients is relevant with regard to a possible prophylactic treatment to prevent those seizures.

The aim of the present study was to assess the independent effect of ES and LS on the long-term mortality after a first-ever supratentorial brain infarct in a large prospective cohort study.

## Patients and methods

All patients aged 18 years and older with a stroke, admitted to the neurology department or seen at the outpatient clinic of Maastricht University Medical Center, are registered in a prospective database; the Maastricht Stroke Registry. For the present study, all patients with a first ever supratentorial brain infarct between July 1st 1987 and September 30th 1990 were included. Brain infarction was defined as the rapid onset of clinical signs of focal cerebral function disturbance, lasting longer than 24 h or leading to death, with no other apparent cause than that of vascular origin, with normal CT or CT showing an area of low attenuation compatible with the clinical signs and symptoms, or autopsy revealing an infarct compatible with the clinical signs and symptoms. When neither CT nor autopsy were available, we used the Guy’s Hospital Stroke Diagnostic Score (Allen score) [22] to determine the probability that the stroke was due to infarction. In this way, 20 patients with an Allen score < 4, i.e. with a probability of 90% or more that their stroke was due to infarction, were also included: 15 of 396 patients without seizures (3.8%), 3 of 16 patients with ES (18.8%) and 2 of 32 patients with LS (6.3%). We excluded patients with previous stroke, clinical signs of cerebellar or brain stem stroke, primary intracerebral haemorrhage, subarachnoid haemorrhage, brain tumour, history of one or more epileptic seizures or a rare cause of the infarction (e.g. vasculitis, arterial dissection, or a hematologic disorder).

We recorded age, gender, clinical infarct syndrome (lacunar or cortical syndrome), degree of disability at stroke onset using the modified Rankin scale (mRS; in the present study used as a surrogate marker for stroke severity), and cardiovascular risk factors, such as smoking, hypertension, diabetes mellitus, and ischaemic heart disease. Clinical infarct syndrome was defined as lacunar or cortical infarct following definitions from the Oxfordshire Community Stroke Project [23]. The cortical infarct group was subdivided in a cardioembolic and atherothrombotic group, depending on the identification of a cardiac embolic source, such as atrial fibrillation (as evidenced by ECG, 24-h heart rhythm monitoring and/or cardiac echocardiography). We defined hypertension, diabetes mellitus and ischemic heart disease as described previously [24]. Data concerning the occurrence of epileptic seizures were collected prospectively by one single investigator (EPM van Raak) during a follow-up period of at least 2 years after inclusion in the Maastricht stroke registry. Seizures during admission were recorded by the treating physician and recorded from the charts by this investigator. After discharge from the hospital, patients were invited for regular follow-up visits at the outpatient clinic every 3–6 months until at least 2 years after the stroke. If patients were unable to attend this visit, the investigator contacted the patient or a close relative or the general practitioner by phone. In case the patient was living in a nursing home the investigator visited the patients every 6 months. Seizure occurrence was recorded when either a focal or a generalized epileptic seizure occurred, excluding other causes of disturbances of consciousness or rhythmic limb movements, such as syncope due to cardiac arrhythmia or limb shaking TIA’s. The date of all seizures and, if possible, the seizure subtypes were registered, as well as eventual antiepileptic drug (AED) treatment. In patients with seizures occurring ≤ 1 week after stroke onset, the seizures were regarded as ES, even when such a patient continued to have seizures after the first week following the initial stroke. In patients with a first seizure occurring > 1 week after stroke onset, the seizures were regarded as LS, as described by the most recent guideline from the International League Against Epilepsy (ILAE) [25]. Only seizures occurring within 2 years after the initial stroke were considered as related to the stroke and regarded as post-stroke seizures. Since most stroke-related seizures do occur within 2 years [2, 8, 26], the etiology of a first seizure occurring more than 2 years after stroke is uncertain; therefore, we did not include these seizures in the present study. In April 2015, the follow-up regarding mortality was conducted retrospectively with end date March 31st 2015. This was done by checking the electronic patient files in our center and the Dutch Municipal Personal Records Database (BRP), the latter being a formal, and reliable national registration of personal details of the entire population of the Netherlands, including birth date and date of death.

### Statistical analysis

Data analysis was performed using SPSS statistics version 23. We compared baseline data with Mann–Whitney *U* test or chi-square test, whichever was appropriate. We assessed mortality using Kaplan–Meier survival curves and determined differences between survival in stroke patients with ES vs. no seizures and in patients with LS vs. no seizures using a log-rank test. We assessed independent contributions to mortality risk with Cox regression analyses with the covariates age at onset of stroke, gender, infarct type (atherothrombotic, cardioembolic or lacunar stroke), mRS as a surrogate marker for stroke severity, hypertension, diabetes mellitus, ischemic heart disease, and smoking. A *p* value < 0.05 was regarded as statistically significant.

## Ethical considerations

According to national legislation at the time of registration, informed consent was not required, as only regularly acquired patient data were recorded. The study protocol on long-term mortality was approved by the medical research ethics committee of the University hospital Maastricht and Maastricht University, and the study was conducted with the ethical standard laid down in the declaration of Helsinki and its later amendments.

## Results

Between July 1st 1987 and September 30th 1990, 475 patients with a first-ever supratentorial brain infarction were registered. For this study on post-stroke seizures and long-term mortality, we excluded 7 patients with a history of epilepsy, 17 with a rare cause of stroke and 7 of whom it was not possible to retrieve if and when they died, because they were not registered in the BRP anymore due to emigration. Of the 444 remaining patients, 48 had one or more epileptic seizures within 2 years following their initial stroke: 16 patients (3.6%) had ES and 32 patients (7.2%) suffered from at least one LS. Five patients with ES continued to have seizures in the first month after the stroke, whereas in the other 11 patients seizures were limited to the first week after the stroke. Three patients with ES had a recurrent stroke with new clinical symptoms (neurological deficit), followed by new ES.

Five out of 32 patients with LS had a recurrent stroke with new clinical symptoms before developing seizures. Four of them had a recurrent brain infarction (proven with CT imaging) followed by LS. The other patient had clinical symptoms of a recurrent stroke and epileptic seizures at the same time, and died before CT scan could be performed.

Fourteen patients with ES (87.5%) were treated with an AED, seven of them were treated with carbamazepine and seven with phenytoin. The remaining two patients with ES (12.5%) did not receive AED treatment (they died after 9 and 21 days, respectively). Of the 32 patients with LS, 22 patients (68.8%) were treated with an AED (17 of them received carbamazepine and 5 phenytoin), whereas 10 patients (31.2%) did not receive AED treatment. Six of them experienced only one single seizure and two of them experienced only two focal seizures and were seizure free thereafter without treatment. The remaining two patients died soon after their first seizure (one patient on the same day, one patient the next day).

Baseline characteristics are shown in Table 1. Patients with ES were significantly older and had a significantly higher initial mRS than patients without seizures. Patients with LS had a significantly higher initial mRS than the 1-week survivors without any seizures and their infarct type was different, with significantly more atherothrombotic strokes and less lacunar strokes in patients with LS.

**Table 1:** Baseline characteristics

	Total	No seizures < 2 years poststroke	Early-onset seizures	*p* value	1-week survivors without seizures < 2 years poststroke	Late-onset seizures	*p* value
No. of patients	444 (100%)	396 (89.2%)	16 (3.6%)		378 (85.1%)	32 (7.2%)	
Male gender	235 (52.9%)	212 (53.5%)	10 (62.5%)	0.48	205 (54.2%)	13 (40.6%)	0.14
Age (mean)	70.6 (SD 12.0)	70.4 (SD 12.1)	77.3 (SD 5.7)	0.01	70.0 (SD 12.1)	69.1 (SD 12.5)	0.83
Age (median)	72.0 (range 26–96)	71.5 (range 26–96)	78.0 (range 65–83)	0.01	71 (range 26–94)	73.5 (range 40–87)	0.83
DM	95 (21.4%)	83 (21.0%)	3 (18.8%)	0.83	78 (20.6%)	9 (28.1%)	0.32
Hypertension	203 (45.7%)	182 (46.0%)	8 (50.0%)	0.75	175 (46.3%)	13 (40.6%)	0.54
IHD	101 (22.7%)	87 (22.0%)	6 (37.5%)	0.15	81 (21.4%)	8 (25.0%)	0.64
Smoking yes	149 (33.6%)	133 (33.6%)	4 (25.0%)	0.48	128 (33.9%)	12 (37.5%)	0.68
Smoking unk	38 (8.6%)	33 (8.3%)	3 (18.8%)	0.15	31 (8.2%)	2 (6.3%)	1.00
mRS 1	8 (1.8%)	8 (2.0%)	0 (0.0%)	1.00	8 (2.1%)	0 (0.0%)	1.00
mRS 2	88 (19.8%)	87 (22.0%)	0 (0.0%)	0.03	87 (23.0%)	1 (3.1%)	0.01
mRS 3	118 (26.6%)	105 (26.5%)	3 (18.8%)	0.77	105 (27.8%)	10 (31.3%)	0.68
mRS 4	97 (21.8%)	90 (22.7%)	3 (18.8%)	1.00	90 (23.8%)	4 (12.5%)	0.14
mRS 5	133 (30.0%)	106 (26.8%)	10 (62.5%)	0.00	88 (23.3%)	17 (53.1%)	0.00
Infarct-type AT	174 (39.2%)	147 (37.1%)	6 (37.5%)	0.98	141 (37.3%)	21 (65.6%)	0.00
Infarct-type CE	102 (23.0%)	88 (22.2%)	7 (43.8%)	0.07	76 (20.1%)	7 (21.9%)	0.81
Infarct-type LACI	168 (37.8%)	161 (40.7%)	3 (18.8%)	0.08	161 (42.6%)	4 (12.5%)	0.00

*DM* diabetes mellitus, *IHD* ischemic heart disease, *Smoking unk* smoking status is unknown, *mRS* modified Rankin scale, *AT* atherothrombotic infarct type, *CE* cardioembolic infarct type, *LACI* lacunar infarct type

Follow-up duration was 24.5–27.8 years (mean 26.0 years, SD 0.9 years). At the end of the follow-up in April 2015, 408 patients had died (91.9%), including all 16 patients with ES (100%) and 29 patients with LS (90.6%). Patients with ES had a significantly shorter survival time than patients without any seizures (*p* = 0.02). Median survival for all 444 patients was 5.2 years (range 1 day–27.7 years), median survival for the 396 patients without seizures was also 5.2 years (range 1 day–27.7 years), whereas median survival for the ES group was 2.2 years (range 10 days–13.6 years) and for the LS group 5.3 years (range 16 days–26.8 years).

### Mortality after early-onset seizures

**Fig. 1:**
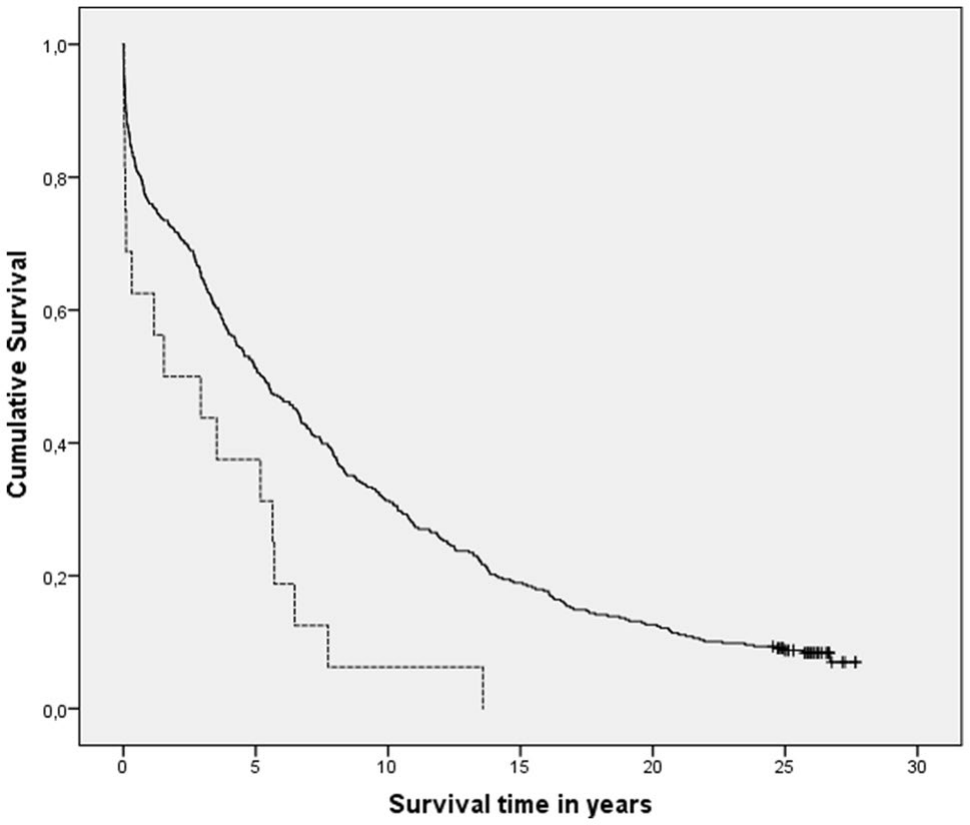
Kaplan–Meier survival curves of patients with early-onset seizures (dotted line) vs. all seizure-free patients (black line); *p* = 0.002

Kaplan–Meier analysis comparing patients with ES (*n* = 16) and patients without any seizures (*n* = 396) showed a statistically significant increase in mortality in patients with ES (*p* = 0.002; Fig. 1). However, Cox regression analysis showed that ES did not influence mortality independently (HR 1.09; 95% CI 0.64–1.85, *p* = 0.76), but that long-term mortality was determined by older age, male gender, diabetes mellitus, infarct type (cardioembolic strokes having higher mortality), stroke severity (higher initial mRS having higher mortality), and smoking status unknown (smoking status was mostly unknown in older patients with high initial mRS) (Table 2). Because of the long follow-up interval, we also determined the influence of ES on mortality at intermediate time-intervals, as in the very long-term some risk factors for mortality may overshadow the effect of ES on mortality. These results are shown in Tables 3 and 4. Univariate Kaplan–Meier analyses (Table 3) showed a significant relation between ES and mortality at 2, 10 and 26 years after stroke, which was lost in the multivariate Cox regression analyses (Table 4). Older age, high initial mRS (and consequently also the age and mRS-related smoking status unknown), and cardioembolic infarct type had a significant relation with mortality at all time points, whereas diabetes mellitus and male gender only played a role in the longer follow-up duration (Table 4).

**Table 2:** Cox regression results

	Early-onset seizures		Late-onset seizures	
	Hazard ratio (95% CI)	*p* value	Hazard ratio (95% CI)	*p* value
Early-onset seizures	1.09 (0.64–1.85)	0.76		
Late-onset seizures			0.81 (0.54–1.20)	0.28
Older age (per year)	1.09 (1.08–1.10)	0.00	1.09 (1.08–1.11)	0.00
Male gender	1.28 (1.01–1.61)	0.04	1.35 (1.07–1.71)	0.01
DM	1.50 (1.16–1.95)	0.00	1.49 (1.15–1.94)	0.00
IHD	1.17 (0.91–1.50)	0.24	1.09 (0.84–1.42)	0.52
Hypertension	1.11 (0.89–1.37)	0.35	1.10 (0.88–1.36)	0.40
Smoking		0.01		0.00
Smoking vs. non-smoking	1.22 (0.95–1.56)	0.12	1.13 (0.88–1.46)	0.33
Smoking unk vs. non-smoking	1.70 (1.17–2.46)	0.01	1.98 (1.35–2.91)	0.00
Smoking unk vs. smoking	1.39 (0.93–2.11)	0.11	1.75 (1.14–2.68)	0.01
mRS		0.00		0.09
mRS 3 vs. 1 or 2	1.14 (0.82–1.58)	0.44	1.19 (0.86–1.64)	0.30
mRS 4 or 5 vs. 1 or 2	1.58 (1.15–2.16)	0.00	1.40 (1.02–1.91)	0.04
mRS 4 or 5 vs. 3	1.38 (1.08–1.77)	0.01	1.18 (0.92–1.50)	0.20
Infarct type		0.00		0.05
Infarct-type AT vs. LACI	1.12 (0.88–1.43)	0.37	1.12 (0.88–1.43)	0.35
Infarct-type CE vs. LACI	1.63 (1.24–2.14)	0.00	1.43 (1.07–1.92)	0.02
Infarct-type CE vs. AT	1.46 (1.11–1.92)	0.01	1.28 (0.96–1.70)	0.10

Note that all analyses were run two times: once with indicator first and once with indicator last, resulting in three different comparisons to report for the variables smoking, mRS and infarct type

*DM* diabetes mellitus, *IHD* ischemic heart disease, *Smoking unk* smoking status is unknown, *mRS* modified Rankin scale, *AT* atherothrombotic infarct type, *CE* cardioembolic infarct type, *LACI* lacunar infarct type

**Table 3:** Kaplan–Meier *p* values for both early-onset seizures and late-onset seizures at several time-intervals

	Early-onset seizures	Late-onset seizures
1 year after the stroke	0.15	NA
2 years after the stroke	0.04	0.79
5 years after the stroke	0.12	0.84
10 years after the stroke	0.00	0.76
26 years after the stroke	0.00	0.72

**Table 4:** Cox regression results for early-onset seizures at several time-intervals

	Follow-up									
	1 year		2 years		5 years		10 years		26 years	
	Hazard ratio (95% CI)	*p* value	Hazard ratio (95% CI)	*p* value	Hazard ratio (95% CI)	*p* value	Hazard ratio (95% CI)	*p* value	Hazard ratio (95% CI)	*p* value
Early-onset seizures	0.96 (0.41–2.25)	0.93	1.14 (0.54–2.41)	0.72	0.83 (0.43–1.63)	0.59	1.02 (0.59–1.78)	0.93	1.09 (0.64–1.85)	0.76
Older age (per year)	1.06 (1.03–1.09)	0.00	1.06 (1.03–1.08)	0.00	1.07 (1.05–1.09)	0.00	1.08 (1.06–1.09)	0.00	1.09 (1.08–1.10)	0.00
Male gender	1.10 (0.69–1.75)	0.68	1.04 (0.68–1.59)	0.85	1.19 (0.86–1.64)	0.30	1.19 (0.91–1.57)	0.21	1.28 (1.01–1.61)	0.04
DM	1.31 (0.81–2.11)	0.28	1.26 (0.81–1.96)	0.30	1.29 (0.92–1.81)	0.15	1.46 (1.10–1.93)	0.01	1.50 (1.16–1.95)	0.00
IHD	1.42 (0.92–2.18)	0.11	1.23 (0.82–1.85)	0.31	1.05 (0.76–1.45)	0.79	1.15 (0.87–1.52)	0.32	1.17 (0.91–1.50)	0.24
Hypertension	0.97 (0.64–1.49)	0.90	0.96 (0.65–1.41)	0.84	1.02 (0.76–1.37)	0.91	0.97 (0.76–1.24)	0.81	1.11 (0.89–1.37)	0.35
Smoking		0.12		0.16		0.07		0.02		0.01
Smoking vs. non-smoking	1.03 (0.61–1.75)	0.91	1.01 (0.62–1.64)	0.97	1.01 (0.70–1.46)	0.95	1.20 (0.90–1.61)	0.21	1.22 (0.95–1.56)	0.12
Smoking unknown vs. non-smoking	1.77 (1.02–3.07)	0.04	1.64 (0.98–2.75)	0.06	1.65 (1.08–2.53)	0.02	1.75 (1.18–2.58)	0.01	1.70 (1.17–2.46)	0.01
Smoking unknown vs. smoking	1.71 (0.87–3.39)	0.12	1.62 (0.86–3.08)	0.14	1.63 (0.98–2.70)	0.06	1.45 (0.93–2.26)	0.10	1.39 (0.93–2.11)	0.11
mRS		0.00		0.00		0.02		0.00		0.00
mRS 3 vs. 1 or 2	1.14 (0.45–2.88)	0.79	1.13 (0.49–2.57)	0.78	1.29 (0.76–2.21)	0.35	1.21 (0.79–1.84)	0.38	1.14 (0.82–1.58)	0.44
mRS 4 or 5 vs. 1 or 2	2.61 (1.12–6.06)	0.03	2.53 (1.20–5.36)	0.02	1.83 (1.11–3.03)	0.02	1.85 (1.25–2.73)	0.00	1.58 (1.15–2.16)	0.00
mRS 4 or 5 vs. 3	2.29 (1.35–3.91)	0.00	2.24 (1.39–3.64)	0.00	1.42 (1.01–1.98)	0.04	1.53 (1.15–2.03)	0.00	1.38 (1.08–1.77)	0.01
Infarct type		0.00		0.00		0.00		0.00		0.00
Infarct-type AT vs. LACI	1.60 (0.93–2.77)	0.09	1.60 (0.98–2.60)	0.06	1.24 (0.88–1.75)	0.23	1.14 (0.86–1.51)	0.36	1.12 (0.88–1.43)	0.37
Infarct-type CE vs. LACI	3.03 (1.75–5.24)	0.00	2.69 (1.64–4.42)	0.00	1.84 (1.28–2.65)	0.00	1.69 (1.24–2.30)	0.00	1.63 (1.24–2.14)	0.00
Infarct-type CE vs. AT	1.89 (1.20–2.97)	0.01	1.69 (1.11–2.56)	0.02	1.49 (1.05–2.10)	0.02	1.48 (1.09–2.00)	0.01	1.46 (1.11–1.92)	0.01

Note that all analyses were run two times: once with indicator first and once with indicator last, resulting in three different comparisons to report for the variables smoking, mRS and infarct type

*DM* diabetes mellitus, *IHD* ischemic heart disease, *mRS* modified Rankin scale, *AT* atherothrombotic infarct type, *CE* cardioembolic infarct type, *LACI* lacunar infarct type

### Mortality after late-onset seizures

**Fig. 2:**
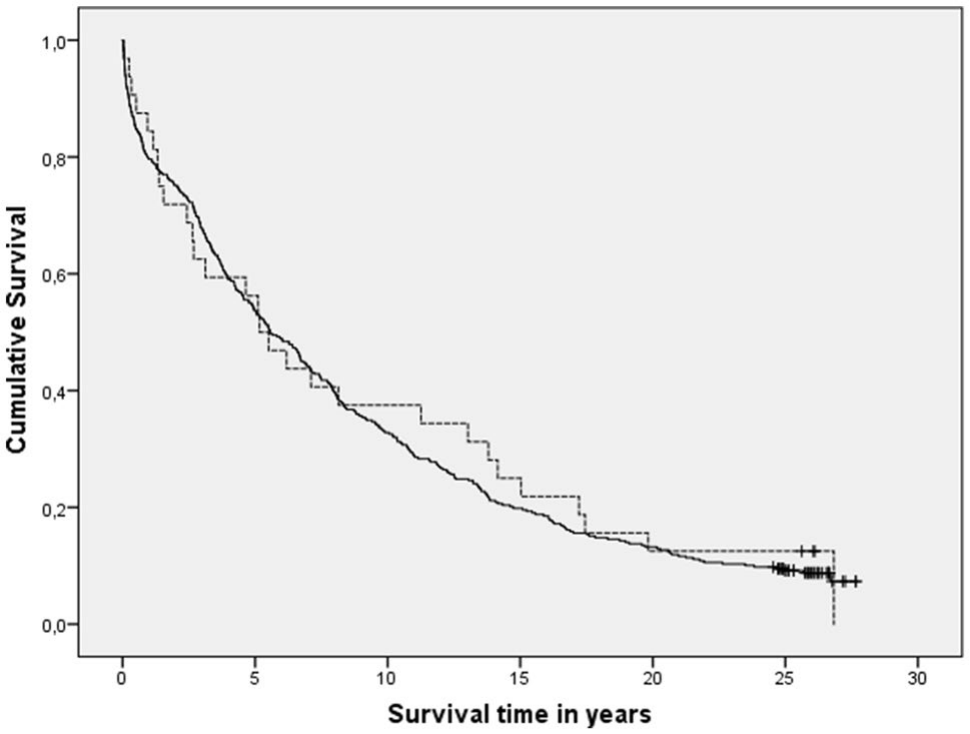
Kaplan–Meier survival curves of patients with late-onset seizures (dotted line) vs. 1-week survivors without any seizures (black line); *p* = 0.717

In the analyses on LS, we included 1-week survivors without seizures (*n* = 378) and patients with LS (*n* = 32). Eighteen patients without any seizures, who died during the first week following their stroke, were excluded as they were not at risk for developing LS. The Kaplan–Meier analysis showed no significant difference in survival (*p* = 0.717; Fig. 2). Cox regression analysis confirmed that LS had no significant influence on mortality (HR 0.81, 95% CI 0.54–1.20, *p* = 0.28), but that long-term mortality was determined by the same risk factors as in the analyses on ES: older age, male gender, diabetes mellitus, smoking status unknown (smoking status was mostly unknown in older patients with high initial mRS), stroke severity (higher initial mRS having higher mortality), and infarct type (cardioembolic strokes having higher mortality) (Table 2). We also determined the influence of LS on mortality at intermediate time-intervals. LS had no significant influence on mortality at all follow-up durations (Tables 3, 5). Similar as in the ES group, older age, cardioembolic infarct type and high initial mRS (and consequently also the age and mRS-related smoking status unknown) had a significant relation with mortality at all time points, whereas diabetes mellitus and male gender only played a role in the longer follow-up duration (Table 5).

**Table 5:** Cox regression results for late-onset seizures at several time-intervals

	Follow-up							
	2 years		5 years		10 years		26 years	
	Hazard ratio (95% CI)	*p* value	Hazard ratio (95% CI)	*p* value	Hazard ratio (95% CI)	*p* value	Hazard ratio (95% CI)	*p* value
Late-onset seizures	0.97 (0.48–1.98)	0.94	0.87 (0.50–1.52)	0.62	0.80 (0.50–1.27)	0.34	0.81 (0.54–1.20)	0.28
Older age (per year)	1.06 (1.03–1.08)	0.00	1.07 (1.05–1.09)	0.00	1.08 (1.06–1.09)	0.00	1.09 (1.08–1.11)	0.00
Male gender	1.33 (0.84–2.11)	0.22	1.38 (0.99–1.94)	0.06	1.29 (0.97–1.71)	0.08	1.35 (1.07–1.71)	0.01
DM	1.17 (0.73–1.89)	0.51	1.22 (0.85–1.73)	0.28	1.47 (1.10–1.96)	0.01	1.49 (1.15–1.94)	0.00
IHD	1.22 (0.78–1.89)	0.39	0.99 (0.70–1.39)	0.95	1.07 (0.80–1.43)	0.65	1.09 (0.84–1.42)	0.52
Hypertension	1.09 (0.73–1.63)	0.69	1.03 (0.76–1.39)	0.85	0.94 (0.73–1.20)	0.61	1.10 (0.88–1.36)	0.40
Smoking		0.06		0.00		0.00		0.00
Smoking vs. non-smoking	0.91 (0.54–1.53)	0.72	0.95 (0.65–1.39)	0.79	1.11 (0.83–1.51)	0.48	1.13 (0.88–1.46)	0.33
Smoking unknown vs. non-smoking	1.91 (1.08–3.38)	0.03	2.14 (1.38–3.32)	0.00	2.14 (1.43–3.22)	0.00	1.98 (1.35–2.91)	0.00
Smoking unknown vs. smoking	2.11 (1.04–4.27)	0.04	2.25 (1.33–3.80)	0.00	1.92 (1.21–3.06)	0.01	1.75 (1.14–2.68)	0.01
mRS		0.01		0.13		0.04		0.09
mRS 3 vs. 1 or 2	1.19 (0.54–2.62)	0.67	1.39 (0.82–2.35)	0.23	1.28 (0.84–1.93)	0.25	1.19 (0.86–1.64)	0.30
mRS 4 or 5 vs. 1 or 2	2.18 (1.05–4.53)	0.04	1.65 (1.00–2.73)	0.05	1.61 (1.09–2.39)	0.02	1.40 (1.02–1.91)	0.04
mRS 4 or 5 vs. 3	1.84 (1.13–3.00)	0.02	1.19 (0.85–1.67)	0.30	1.26 (0.95–1.68)	0.10	1.18 (0.92–1.50)	0.20
Infarct type		0.07		0.08		0.05		0.05
Infarct-type AT vs. LACI	1.17 (0.72–1.92)	0.52	1.11 (0.78–1.58)	0.55	1.11 (0.84–1.47)	0.47	1.12 (0.88–1.43)	0.35
Infarct-type CE vs. LACI	1.82 (1.07–3.10)	0.03	1.55 (1.05–2.30)	0.03	1.50 (1.08–2.09)	0.02	1.43 (1.07–1.92)	0.02
Infarct-type CE vs. AT	1.55 (0.95–2.52)	0.08	1.39 (0.96–2.03)	0.08	1.35 (0.98–1.97)	0.07	1.28 (0.96–1.70)	0.10

Note that all analyses were run two times: once with indicator first and once with indicator last, resulting in three different comparisons to report for the variables smoking, mRS and infarct type

*DM* diabetes mellitus, *IHD* ischemic heart disease, *mRS* modified Rankin scale, *AT* atherothrombotic infarct type, *CE* cardioembolic infarct type, *LACI* lacunar infarct type

## Discussion

In this study, we showed that both ES and LS did not influence long-term mortality after ischemic stroke. We found that patients with ES had a higher mortality; however, this effect was due to the confounding factors (older age, male gender, diabetes mellitus, infarct type and stroke severity as measured by the mRS) and not to the seizures themselves as shown in the multivariate analysis. Most other studies also found no independent relation between ES and higher mortality [14–18]. The relationship between ES and mortality remained significant after correction for confounders in studies by Arboix et al. [19, 20]. Stroke patients suffer by definition from a cardiovascular disease and, therefore, are at an increased risk to develop another cardiovascular disease and consequently have an increased mortality [27]. Given this increased risk of mortality due to vascular disease it is more difficult to distinguish an eventual increase in mortality due to early and late seizures, which might explain the conflicting results found in different studies.

In our study, LS had no significant influence on survival in univariate nor multivariate analyses. This contrasts with the study by Arntz et al. [21], which showed an increased long-term mortality in patients with stroke at young age and post-stroke epilepsy. The seemingly opposite outcome of our study can be explained mostly by differences in study population and study design. In the study by Arntz et al. only younger patients (aged 18–50 years) with a TIA or ischemic stroke were included, and the occurrence of seizures was determined retrospectively, which may have led to a recall bias. Also, all seizures which occurred poststroke were included, also when these occurred more than 2 years after the index TIA or stroke. In our study only 33 patients aged ≤ 50 years were included, none of those developed ES, and only 4 of the younger patients developed LS. These four patients all had severe strokes (initial mRS 4 or 5). Our young stroke patients with LS had a survival of 75% after 26 years vs. 79% in our young patients without LS (non-significant, data not shown). Mortality in our young stroke patients was also not influenced by LS in a tentative Cox-regression analysis (data not shown). In the study by Arntz et al. the significant effect of post-stroke seizures on mortality was only found in patients with mild stroke (NIHSS 0–4). They found no significant correlation between post-stroke seizures and mortality in patients with moderate stroke, nor in those with severe stroke. Therefore, our results in the young stroke patients are in line with the result from the study by Arntz et al., since we also found no correlation between LS and long-term mortality in young stroke patients with severe strokes.

When comparing our data to other studies about incidence of seizures in stroke patients, we notice a larger part of lacunar stroke in our seizure patients [8, 28]. We hypothesize that these seizures may be caused by microvascular changes in the brain, including cortical parts of the brain, and not by the lacunar stroke itself, as also suggested by de Reuck et al. and Arboix et al. [29–31].

Strengths of our study are our method of selection and follow-up. In our study data regarding the occurrence of seizures were gathered prospectively for two consecutive years by one single investigator. Considerable effort was put into gathering information about the occurrence of possible seizures. Therefore, we think our recall bias is low. Also, we chose to include only patients with first ever supratentorial brain infarct and only seizures which occurred within 2 years after the first-ever stroke, which means only seizures which are likely to be ascribed to the patient’s stroke were taken into account. Furthermore, our long-term mortality follow-up and a statistical analysis with correction for known risk factors for (long term) mortality are major strengths of this study and make it possible to make a firm statement on the relation between poststroke seizures and long term mortality.

Our study has some limitations. First, not all patients underwent CT scanning; therefore, some patients may have had a haemorrhagic stroke. Since haemorrhagic stroke patients have a higher risk of poststroke seizures [2, 32], patients with haemorrhagic stroke may be overrepresented in our ES group. This might have influenced mortality rates, since mortality is higher in patients with haemorrhagic stroke [33]. However, we believe this effect to be small, as we performed all analyses anew excluding the 20 patients without CT-scanning and found the same results (data not shown). Second, we used a surrogate marker for stroke severity, the initial mRS, which actually is an outcome scale and a crude assessment of function directly after a stroke. As the NIHSS was not available yet at the time of inclusion start and no other stroke severity score was recorded, we unfortunately could not use a more reliable measure for stroke severity.

A third limitation is the difference in group size, where the seizure groups are small as compared to the stroke group, which makes it more difficult to detect a clinically relevant difference in mortality. Last, our follow-up of 2 years with regard to the development of seizures might have excluded some patients who developed seizures at a later time point. Though the incidence of new seizures more than 2 years after a stroke is low, we will certainly have missed some events.

The authors of the FUTURE study [21] state that future studies should investigate the role of antiepileptic drugs in preventing post-stroke epilepsy. However, this would only be true for the specific young stroke population they studied, since in our study the relation between LS and mortality was not significant. Before starting studies aimed at prophylactic treatment with antiepileptic drugs in stroke patients, more effort should be done to elaborate the exact influence of LS in stroke patients, not only with regard to mortality but also concerning the effect on stroke recovery and quality of life [34].

In conclusion, neither ES nor LS have a negative impact on long-term mortality in ischemic stroke patients. Mortality is, therefore, no argument to support the prophylactic use of antiepileptic drugs to prevent seizures in ischemic stroke patients. More research is necessary on the impact of especially LS on mortality, neurologic recovery from stroke and quality of life.

## References

[1] Alberti A, Paciaroni M, Caso V, Venti M, Palmerini F, Agnelli G (2008). Early seizures in patients with acute stroke: frequency, predictive factors, and effect on clinical outcome. Vasc Health Risk Manag.

[2] Bladin CF, Alexandrov AV, Bellavance A (2000). Seizures after stroke: a prospective multicenter study. Arch Neurol.

[3] De Herdt V, Dumont F, Henon H (2011). Early seizures in intracerebral hemorrhage: incidence, associated factors, and outcome. Neurology.

[4] Rossi C, De Herdt V, Dequatre-Ponchelle N, Henon H, Leys D, Cordonnier C (2013). Incidence and predictors of late seizures in intracerebral hemorrhages. Stroke.

[5] Procaccianti G, Zaniboni A, Rondelli F, Crisci M, Sacquegna T (2012). Seizures in acute stroke: Incidence, risk factors and prognosis. Neuroepidemiology.

[6] Lancman ME, Golimstok A, Norscini J, Granillo R (1993). Risk factors for developing seizures after a stroke. Epilepsia.

[7] Zhang C, Wang X, Wang Y (2014). Risk factors for post-stroke seizures: a systematic review and meta-analysis. Epilepsy Res.

[8] Burn J, Dennis M, Bamford J, Sandercock P, Wade D, Warlow C (1997). Epileptic seizures after a first stroke: the Oxfordshire community stroke project. BMJ.

[9] Wang JZ, Vyas MV, Saposnik G, Burneo JG (2017). Incidence and management of seizures after ischemic stroke: systematic review and meta-analysis. Neurology.

[10] Ferro JM, Pinto F (2004). Poststroke epilepsy: epidemiology, pathophysiology and management. Drugs Aging.

[11] Berges S, Moulin T, Berger E (2000). Seizures and epilepsy following strokes: recurrence factors. Eur Neurol.

[12] Fisher RS, Acevedo C, Arzimanoglou A (2014). ILAE official report: a practical clinical definition of epilepsy. Epilepsia.

[13] Thurman DJ, Logroscino G, Beghi E (2017). The burden of premature mortality of epilepsy in high-income countries: a systematic review from the mortality task force of the international league against epilepsy. Epilepsia.

[14] Reith J, Jorgensen HS, Nakayama H, Raaschou HO, Olsen TS (1997). Seizures in acute stroke: Predictors and prognostic significance. The Copenhagen Stroke Study. Stroke.

[15] Labovitz DL, Hauser WA, Sacco RL (2001). Prevalence and predictors of early seizure and status epilepticus after first stroke. Neurology.

[16] Hamidou B, Aboa-Eboule C, Durier J (2013). Prognostic value of early epileptic seizures on mortality and functional disability in acute stroke: the Dijon Stroke Registry (1985–2010). J Neurol.

[17] Szaflarski JP, Rackley AY, Kleindorfer DO (2008). Incidence of seizures in the acute phase of stroke: a population-based study. Epilepsia.

[18] Davalos A, De Cendra E, Molins A, Ferrandiz M, Lopez-Pousa S, Genis D (1992). Epileptic seizures at the onset of stroke. Cerebrovasc Dis.

[19] Arboix A, Comes E, Massons J, Garcia L, Oliveres M (1996). Relevance of early seizures for in-hospital mortality in acute cerebrovascular disease. Neurology.

[20] Arboix A, Comes E, Garcia-Eroles L, Massons JB, Oliveres M, Balcells M (2003). Prognostic value of very early seizures for in-hospital mortality in atherothrombotic infarction. Eur Neurol.

[21] Arntz RM, Rutten-Jacobs LC, Maaijwee NA (2015). Poststroke epilepsy is associated with a high mortality after a stroke at young age: follow-up of transient ischemic attack and stroke patients and unelucidated risk factor evaluation study. Stroke.

[22] Allen CM (1983). Clinical diagnosis of the acute stroke syndrome. Q J Med.

[23] Bamford J, Sandercock P, Jones L, Warlow C (1987). The natural history of lacunar infarction: the Oxfordshire Community Stroke Project. Stroke.

[24] de Jong G, van Raak L, Kessels F, Lodder J (2003). Stroke subtype and mortality. a follow-up study in 998 patients with a first cerebral infarct. J Clin Epidemiol.

[25] Beghi E, Carpio A, Forsgren L (2010). Recommendation for a definition of acute symptomatic seizure. Epilepsia.

[26] Lossius MI, Ronning OM, Slapo GD, Mowinckel P, Gjerstad L (2005). Poststroke epilepsy: occurrence and predictors—a long-term prospective controlled study (Akershus Stroke Study). Epilepsia.

[27] Achterberg S, Cramer MJ, Kappelle LJ (2010). Patients with coronary, cerebrovascular or peripheral arterial obstructive disease differ in risk for new vascular events and mortality: the SMART study. Eur J Cardiovasc Prev Rehabil.

[28] Stefanidou M, Das RR, Beiser AS (2017). Incidence of seizures following initial ischemic stroke in a community-based cohort: The Framingham Heart Study. Seizure.

[29] De Reuck J, Nagy E, Van Maele G (2007). Seizures and epilepsy in patients with lacunar strokes. J Neurol Sci.

[30] De Reuck J, Van Maele G (2009). Cognitive impairment and seizures in patients with lacunar strokes. Eur Neurol.

[31] Arboix A, Blanco-Rojas L, Marti-Vilalta JL (2014). Advancements in understanding the mechanisms of symptomatic lacunar ischemic stroke: translation of knowledge to prevention strategies. Expert Rev Neurother.

[32] Beghi E, D’Alessandro R, Beretta S (2011). Incidence and predictors of acute symptomatic seizures after stroke. Neurology.

[33] Andersen KK, Olsen TS, Dehlendorff C, Kammersgaard LP (2009). Hemorrhagic and ischemic strokes compared: stroke severity, mortality, and risk factors. Stroke.

[34] Sykes L, Wood E, Kwan J (2014). Antiepileptic drugs for the primary and secondary prevention of seizures after stroke. Cochrane Database Syst Rev.

