# Correction to: The occurrence of seizures after ischemic stroke does not influence long-term mortality; a 26-year follow-up study

**DOI:** 10.1007/s00415-018-9020-7

**Published:** 2018-08-31

**Authors:** J. H. van Tuijl, E. P. M. van Raak, R. J. van Oostenbrugge, A. P. Aldenkamp, R. P. W. Rouhl

**Affiliations:** 1 Department of Neurology, Maastricht University Medical Center, PO Box 5800, 6202 AZ Maastricht, The Netherlands; 2 Department of Neurology, Elisabeth-Tweesteden Hospital, Tilburg, The Netherlands; 3 School for Mental Health and Neurosciences, Maastricht University, Maastricht, The Netherlands; 4 Cardiovascular Research Institute Maastricht (CARIM), Maastricht University Medical Center, Maastricht, The Netherlands; 5 Academic Center for Epileptology, Maastricht University Medical Center and Kempenhaeghe Center of Expertise for Epileptology, Maastricht, The Netherlands; 6 Faculty of Electrical Engineering, University of Technology, Eindhoven, The Netherlands

## Correction to: Journal of Neurology (2018) 265:1780–1788 10.1007/s00415-018-8907-7

The original version of this article unfortunately contained a mistake. The copyright line was incorrect in the HTML version. The correct copyright line should be “The Author(s) 2018”.

